# Suffering and hope: Helen Lester Memorial Lecture 2016

**DOI:** 10.3399/bjgpopen17X100605

**Published:** 2017-02-15

**Authors:** Christopher Dowrick

**Affiliations:** Professor of Primary Medical Care, Institute of Psychology Health and Society, University of Liverpool, Liverpool, UK

**Keywords:** consultation, general practice, hope, primary care, stress psychological, suffering

## Introduction

In her magnificent 2012 James Mackenzie Lecture, Helen Lester directly challenged the persisting stigma of mental illness, calling us all to change from being bothered by to bothering about Billy, a homeless, alienated human being living with psychosis. She talked passionately about her drop-in medical service for the homeless of Birmingham, where she heard stories of unemployment and divorce, of childhood traumas and teenage breakdowns, and how she was *‘... left with an unshakeable impression that psychosis was something that primary care could and should engage with.’^1^*

Helen demonstrated in this lecture, and in her life’s work, two themes that are fundamental to medicine in general,^2^ and to primary mental health care in particular: acknowledging suffering and offering hope. These are the themes that I wish to discuss, in her memory, this evening.

I will explore our ambivalence to acknowledging suffering, how despite our best intentions we often find it hard to really listen to our patient’s distress, and I will suggest some things that might help us to listen better. I will then discuss how we can offer hope: through compassion, by being thoughtfully positive, from the discovery and application of new knowledge, and perhaps by changing the ways in which we think about ourselves and our patients.

### Acknowledging suffering

#### Listening

When presented with suffering, the first thing we need to do is to listen. In the words of psychoanalyst Wilfred Bion, we should be listening *without memory or desire*: when we listen with memory, we are intent on making the speaker part of an old agenda; when we listen with desire we are intent on making them part of a new one.

That suffering may be expressed in poetic form, as composed here in Dublin by Gerard Manley Hopkins:

‘No worst, there is none. Pitched past pitch of grief
More pangs will, schooled at forepangs, wilder ring […]
O the mind, mind has mountains; cliffs of fall
Frightful, sheer, no-man-fathomed’.^3^

Or in the paintings of Francis Bacon, portraying his subjects enclosed in the wretched glass capsule of the human individual.

Or in the collective sorrow witnessed by WB Yeats, when ‘*Things fall apart; the centre cannot hold;* [and] *Mere anarchy is loosed upon the world’*:^4^ be it the continuing aftermath of the Iraq war, or migration forced by political turmoil in Syria and Libya.

Or in the bristling, frustrated anguish of 19-year-old Darren, piercings through lip, nose, eyebrows, and scarring up both arms. He tells me about parental separation, fostering and sexual abuse, bullying in school, and how booze keeps him from feeling too much but leads to fights with friends, nightclub doormen, and the police. His only comfort is beating the hell out of his drum kit in the middle of the night. He does not think he will live much longer, and I fear he may be right.

#### Shying away from suffering

To listen purely, to just listen, is the most valuable thing. But it is also the most difficult thing. Ian McWhinney warns that our ‘... *lack of openness in the face of suffering* [...] *closes off compassion and stops us from being healers.’* ^5^

There are many ways in which we try to distance ourselves from suffering.

We may dismiss the patient’s problem, offer rudimentary reassurance or provide simplistic explanations unrelated to his concerns.^6^

We may reach for a diagnosis as a form of emotional safety blanket, trying to turn raw human suffering (unbearable by the doctor) into a disease (something treatable, therefore bearable by the doctor).^7^ This helps to explain why GPs often over-diagnose and over-treat depression.^8,9^

We create even more distance when we introduce the diagnosis of depression into cross-cultural consultations. The *C*ross-Cultural RE-ORDER study, involving GPs and people of Vietnamese and East Timorese origin in Melbourne,^10 ^posed a central dilemma: how to integrate experiences grounded in one social context within the matrices provided by another? We identified a *tremendous collision* between migrants, whose experience was framed by patterns of alienation and traumatised self-identity, and GPs, who interpreted distress as technical problems of practice. In this context, the diagnosis of depression was not a clinical entity, but a mechanism of decoupling: replacing loss with illness, it individualised previously social problems. In doing so, the GPs were effectively distancing themselves from the reality of the suffering being presented.

The systems within which we operate may also negate our openness to suffering.^11^ Communication training emphasises cognitive aspects of our patients’ experiences — their ideas, concerns, and expectations — but neglects the crucial question of what they may be feeling. And being open to suffering becomes harder as annual consultation rates rise and continuity of care declines, accompanied by the increasing emphasis on protocol-driven care,^12^ the relentless hunt for Quality and Outcomes Framework (QOF) points and the distracting omnipresence of the computer in the consulting room.^13^

#### Turning toward suffering

Ronald Epstein recommends that we turn toward suffering: actively seek to recognise it, become curious about the patient’s experience, and intentionally become more present and engaged.^14^ It’s not simply a question of deciding to do this: if it is our intention, then we probably need to be enabled. Mentors, peer support, and supervision are all important. Formal training is also beneficial: it increases empathy and patient-centred care, reduces burnout and enhances our adaptive reserves.^15,16^

#### Sunt lacrimae rerum

Bearing witness to suffering, giving our patients a sense of being understood and accepted, is the first essential^17,18^ step towards hope.

In the first book of Virgil’s *Aeneid*, Aeneas is in Carthage, seeking refuge from the ravages of the Trojan war. Gazing at a mural in a temple, depicting the deaths of many of his friends and countrymen, he is moved to tears, and offers a rousing tribute including the words *sunt lacrimae rerum.*

I propose that this phrase brings us to the heart of the link between suffering and hope.

*Rerum* is the genitive of *res* (things) and can be understood in both objective and subjective terms. This phrase has been translated as either ‘there are tears for things’, or else ‘there are tears of things’. The first, objective version indicates the burdens we have to bear, the frailty of human existence, and the ‘shit-life syndrome’ so many patients experience. The second, subjective version indicates that things feel sorrow for our suffering; that in some sense the universe feels our pain.

Virgil is aware of the ambiguity and wishes to us to understand both meanings at the same time. So does Seamus Heaney, who translates the phrase as ‘There are tears at the heart of things.’^19^

This is its richness and power in the consultation. When I experience and express compassion for the suffering of the person in the room with me, both senses of *sunt lacrimae rerum* are in play. My patient can express pain, distress, and suffering, knowing that, from me, he finds understanding, compassion, and safety. My consulting room has become, momentarily, a sanctuary.

Sometimes bearing witness to suffering in the face of overwhelming life difficulties may be all that is possible, or necessary. Listening to Darren, behind his angry ranting I hear a lost, lonely, frightened little boy. I want to give him a hug and bring him home with me, but I content myself with a smile, a handshake, and an agreement to meet again soon.

There is emerging evidence of the beneficial effects of clinical compassion in primary care, across a range of conditions. Patient perceptions of GP empathy are crucial for enablement, which leads to improvement in symptom severity and related wellbeing.^20^ GP empathy is also associated with greater effectiveness of antidepressant medication^21^ and with lower rates of acute metabolic complications in diabetic patients.^22^

### Offering hope

#### Being positive

The next step on the way out of the dark woods in which patients find themselves, is for GPs to be positive, to express hope and optimism. However, as I have noted in a previous discussion paper,^23^ there are several caveats here.

If optimism is expressed too early in the consultation, before the patient has had adequate opportunity to tell his story, this may be perceived as evidence of a lack of empathy or understanding.^24^

There are at least four ways in which the doctor might propose a positive approach. The doctor may indicate that they are an expert in this problem and can solve it for the patient. They may indicate that doctor and patient can successfully work on the problem together; or that the patient has the resources to manage it themselves; or that symptoms will resolve spontaneously without the need for intervention. These all convey hope and optimism but they are very different in their orientation, and it is not clear which will be more effective.

The benefit of a given approach may be affected by the patient’s understanding of the nature of their problem. A doctor-focused approach might be more helpful for someone with a disease-based understanding of their problem, while a time-focused approach might be more helpful for someone who sees themselves as having problems with life’s circumstances.^25^

In any event, we may assume that shaping the patient’s story in a more hopeful direction is likely to be valuable. Talking with Darren, I aim to build on his strengths: his obvious intelligence and his drumming skills.

#### Evidence-based hopefulness

This leads to the third element in our offer of hope. It must be based on the best available evidence. As primary care academics, we are in a unique position to generate and apply evidence about effective primary mental health care.

Below are five hopeful things we now know about primary mental health care.

We have greatly increased our understanding of physical, psychological, and social factors influencing the trajectory of mental health problems presenting in primary care.^26,27^ We can now use this knowledge to offer more accurate prognoses, and tailor interventions to meet patients’ specific needs more effectively. So for Darren, who I know is in a high risk group, I commit myself to seeing him regularly, and offer him the options of psychotropic medication and a referral to our local community mental health team.

We have a much clearer idea of how GPs can communicate therapeutically with patients presenting with unexplained physical symptoms, following their cues and requests for explanation and support, and providing explanations that are tangible, exculpating, and involving.^28^

We know that people with severe and enduring mental illness have reduced life expectancy, due mainly to their increased risk of physical diseases, and that primary care has a pivotal role in redressing this major health inequality.^29^ The Lester Toolkit^30^ is an invaluable resource to promote cardio-metabolic health for patients with psychosis.

We know that active collaboration between primary and secondary care improves access to interventions for untreated psychosis^31^ and improves outcomes for depression.^32^

We know that the success of strategies to increase equity of access to primary mental health care for marginalised groups is critically dependent on mutual engagement between primary care and community stakeholders.^33,34^

Of course there is more evidence-based hopefulness that we still need to generate.

Although the Lester Toolkit and the QOF are enabling equality of access to chronic disease monitoring in primary care for people with severe and enduring mental illness, there remains the challenge of achieving equity of treatment, in order to improve life expectancy for these patients.^35^

We know from the THREAD trial that treatment with an selective serotonin reuptake inhibitor plus supportive care is more effective than supportive care alone for patients with mild to moderate depression in primary care,^36^ but we do not yet know how much of the difference is due to placebo effects.

There is evidence of improved outcomes among patients who take an active approach to managing their own mental health problems: holding illness beliefs which emphasise the benefits of exercise or activity,^37^ or deploying strategies to expand their inner resources.^38^ These associations are intriguing and deserve further investigation.

#### Persons leading lives

My fourth ingredient for an effective offer of hope is concerned with whether and how we think of patients as persons.

An analysis of the metaphors used by GPs in routine consultations showed how we tend to use mechanical metaphors to explain diseases, consider patient’s problems as puzzles, and cast GPs in the roles of problem solvers and controllers of disease.^39^ But there is a fundamental flaw with this perspective. It puts the onus on doctors to achieve change, while undermining patients’ sense of enablement. It is a recipe for burnout and failure, contradicting our intentions of promoting patient self-management and joint decision making.

We should remind ourselves that our patients have creative capacity, that they are capable of leading their own lives and of finding meaning in purposeful engagement with the world around them.^40^ What would be the impact if we generate a new set of dynamic metaphors, offering concepts of the self which contain hope, action and purpose; if I see Darren not as an unemployable borderline personality disorder, but as a critically creative musician?

#### A meadow of delight

Darren has been coming to see me every few weeks for quite a while. He is still alive. He still mostly rants, and I still mostly listen, but there are less booze and fewer fights in his life. He has a girlfriend and a dog, and his drumming skills have found outlet in two local bands, one with a possible recording contract. We’re both beginning to feel more hopeful.

As well as taking the best possible care of our patients, we also need to take good care of ourselves. Whether it’s in the midst of a frantic Friday afternoon surgery, a high profile research project where recruitment rates are way behind target, a demand to pack yet more students into an already pressured teaching programme, or an awareness of our own frailty and mortality in the face of traumatic accident or life-threatening disease, we do well to recognise our own suffering and give ourselves the freedom to hope.

I leave you with these words from John O’Donohue:

… when your eyes freeze behind the grey window and the ghost of loss gets in to you, may a flock of colours, indigo, red, green, and azure blue come to awaken in you a meadow of delight.[…] And so may a slow wind work these words of love around you, an invisible cloak to mind your life.^41^
